# Supplementary material for: Daily accumulation rates of floating debris and attached biota on continental and oceanic island shores in the SE Pacific: testing predictions based on global models
Source: PeerJ. 2023 Jul 27;11:e15550. doi: 10.7717/peerj.15550 (PMC10387232; doi:10.7717/peerj.15550)
Supplement: Table S1 — Buoyancy was tested by submerging each item in sea water (for details see Material & Methods). N, number of items. n.d., not determined. Items from quantitative and opportunistic samplings are considered. [file peerj-11-15550-s001.docx]

**Table S1.** Buoyancy of items by region. Buoyancy was tested by submerging each item in sea water (for details see material & methods). N = number of items. n.d. = not determined. Items from quantitative and opportunistic samplings are considered.

| Region | Beach | N (items) | Buoyancy [%] | | | |
| --- | --- | --- | --- | --- | --- | --- |
|  |  |  | positive | negative | unclear | not tested |
| Oceanic:  Rapa Nui | Anakena | 499 | 22 | 0 | 0 | 78 |
|  | Ovahe | 240 | 18 | 0 | 0 | 82 |
| Continental:  South | Mar Brava South | 887 | 87 | 3 | 2 | 8 |
|  | Mar Brava North | 501 | 83 | 1 | 16 | 0 |
| Continental:  Center | Ritoque | 5699 | 95 | 1 | 4 | 0 |
|  | Maitencillo | 2758 | 90 | 1 | 9 | 0 |
| Continental:  North | Choros | 949 | n.d. | n.d. | n.d. | 100 |
