# Supplementary material for: Daily accumulation rates of floating debris and attached biota on continental and oceanic island shores in the SE Pacific: testing predictions based on global models
Source: PeerJ. 2023 Jul 27;11:e15550. doi: 10.7717/peerj.15550 (PMC10387232; doi:10.7717/peerj.15550)
Supplement: Table S2 — n, number of items tested in each category. Buoyancy: positive (item floats), negative (item sinks), or n.d., not determined (not tested or result unclear). [file peerj-11-15550-s002.docx]

**Table S2**. Buoyancy by category. n = number of items tested in each category. Buoyancy: positive (item floats), negative (item sinks), or n.d. = not determined (result unclear). nf = not foamed. *see Fig. S6 for examples of “rope bundles”. PW = processed wood

| **Category** | **n** | **Buoyancy** | | |
| --- | --- | --- | --- | --- |
|  |  | **positive [%]** | **n.d. [%]** | **negative [%]** |
| HPL fragments, not PET | 966 | 99.1 | 0.3 | 0.6 |
| Caps & lids | 705 | 99.9 | 0.1 | 0.0 |
| Lollipop and cottonbud sticks | 383 | 99.7 | 0.0 | 0.3 |
| Other, pooled | 364 | 91.2 | 4.7 | 4.1 |
| **∑ Hard Plastics (HPL)** | **2418** |  |  |  |
|  |  |  |  |  |
| Food wrappers, packaging | 4208 | 96.4 | 0.2 | 3.4 |
| Bags | 324 | 91.7 | 3.4 | 4.9 |
| Cups (nf), single-use | 86 | 100.0 | 0.0 | 0.0 |
| Drinking straws, single-use | 95 | 100.0 | 0.0 | 0.0 |
| Food containers (nf) & cutlery, single-use | 105 | 87.6 | 1.9 | 10.5 |
| Other, pooled | 63 | 85.7 | 6.3 | 7.9 |
| **∑ Thin Plastics (TPL)** | **4881** |  |  |  |
|  |  |  |  |  |
| Foamed food containers, single-use | 281 | 100.0 | 0.0 | 0.0 |
| Foamed plastics, other | 162 | 97.5 | 0.0 | 2.5 |
| Sanitary masks, single-use | 55 | 98.2 | 1.8 | 0.0 |
| Sanitary & medical, other | 82 | 50.0 | 0.0 | 50.0 |
| PET fragments | 26 | 0.0 | 3.8 | 96.2 |
| Other, pooled | 185 | 78.9 | 1.1 | 20.0 |
| **∑ Other Plastics (OPL)** | **843** |  |  |  |
|  |  |  |  |  |
| PW, Ice cream sticks & cutlery | 292 | 100.0 | 0.0 | 0.0 |
| Processed wood, other | 112 | 94.6 | 4.5 | 0.9 |
| Paper, Cardboard & Tetrapack | 119 | 32.8 | 0.0 | 67.2 |
| Organic/ Food rests | 93 | 77.4 | 3.2 | 19.4 |
| Metal | 70 | 27.1 | 1.4 | 71.4 |
| Textile, Shoes, Canvas | 66 | 42.4 | 7.6 | 50.0 |
| Rubber | 49 | 95.9 | 0.0 | 4.1 |
| Glass & Ceramics | 17 | 17.6 | 0.0 | 82.4 |
| Wax | 16 | 100.0 | 0.0 | 0.0 |
| Other, pooled | 9 | 22.2 | 0.0 | 77.8 |
| **∑ Other, Mix** | 975 |  |  |  |
|  |  |  |  |  |
| Ropes and rope bundles | 846 | 97.6 | 1.9 | 0.5 |
| Rope bundles | 77 | 35.1 | 2.6 | 62.3 |
| Ropes | 52 | 80.8 | 13.5 | 5.8 |
| **∑ Ropes, synthetic** | **791** |  |  |  |
|  |  |  |  |  |
| **∑ All items** | **9908** |  |  |  |
