# Supplementary material for: Daily accumulation rates of floating debris and attached biota on continental and oceanic island shores in the SE Pacific: testing predictions based on global models
Source: PeerJ. 2023 Jul 27;11:e15550. doi: 10.7717/peerj.15550 (PMC10387232; doi:10.7717/peerj.15550)
Supplement: Table S3 — (a) For all AMD, (b) for items with pelagic epibionts only. [file peerj-11-15550-s003.docx]

**Table S3**. Arrival rates of litter items [km^-1^] by sampling day, as well as mean ± standard deviation (∅) per beach and region. a) For all AMD, b) for items with pelagic epibionts only.

| 1. **All AMD items [km^-1^]** | | | | | | | |
| --- | --- | --- | --- | --- | --- | --- | --- |
|  | **SPSG_Rapa Nui** | | **Conti_South** | | **Conti_Center** | | **Conti_North** |
| **Day** | **Anakena** | **Ovahe** | **MBS** | **MBN** | **Ritoque** | **Maitencillo** | **Choros** |
| **1** | 360.0 | 337.5 | 105.0 | 50.0 | 124.0 | 31.0 | 68.0 |
| **2** | 228.0 | 87.5 | 137.0 | 80.0 | 114.0 | 269.0 | 94.0 |
| **3** | 284.0 | 425.0 | 146.0 | 25.0 | 79.0 | 130.0 | 128.0 |
| **4** | 236.0 | 325.0 | 61.0 | 45.0 | 69.0 | 93.0 | 64.0 |
| **5** | 192.0 | 75.0 | 39.0 | 76.0 | 167.0 | 87.0 | 76.0 |
| **6** | 216.0 | 500.0 | 84.0 | 27.0 | 94.0 | 56.0 | 91.0 |
| **7** | 188.0 | 375.0 | 66.0 | 43.0 | 646.0 | 20.0 | 369.0 |
| **8** | 160.0 | 350.0 | 33.0 | 25.0 | 1703.0 | 973.0 | 300.0 |
| **9** | 44.0 | 375.0 | 30.0 | 26.0 | 1063.0 | 528.0 | 565.0 |
| **10** | 88.0 | 150.0 | 63.0 | 20.0 | 1090.0 | 270.0 | 9.0 |
| **11** | nd | nd | 48.0 | 16.0 | 650.0 | 340.0 | 18.0 |
| **12** | nd | nd | 75.0 | 16.0 | 444.0 | 262.0 | 27.0 |
|  |  |  |  |  |  |  |  |
| **∅ (Beach)** | 199.6  ± 90.5 | 300.0  ± 145.2 | 73.9  ± 38.2 | 37.4  ± 22.0 | 520.3  ± 529.5 | 254.9  ± 271.8 | 150.8  ± 171.4 |
| **∅ (Region)** | 249.8 ± 128.5 | | 55.7 ± 35.8 | | 387.6 ± 433.3 | | 150.8 ± 171.4 |
|  |  |  |  |  |  |  |  |
| 1. **Items with pelagic epibonts only [km^-1^]** | | | | | | | |
|  | **SPSG_Rapa Nui** | | **Conti_South** | | **Conti_Center** | | **Conti_North** |
| **Day** | **Anakena** | **Ovahe** | **MBS** | **MBN** | **Ritoque** | **Maitencillo** | **Choros** |
| **1** | 68.0 | 37.5 | 1.0 | 1.0 | 1.0 | 0.0 | 0.0 |
| **2** | 56.0 | 0.0 | 0.0 | 1.0 | 0.0 | 0.0 | 0.0 |
| **3** | 100.0 | 75.0 | 0.0 | 0.0 | 1.0 | 2.0 | 0.0 |
| **4** | 88.0 | 62.5 | 0.0 | 0.0 | 2.0 | 0.0 | 0.0 |
| **5** | 20.0 | 12.5 | 0.0 | 0.0 | 2.0 | 1.0 | 0.0 |
| **6** | 48.0 | 62.5 | 0.0 | 0.0 | 1.0 | 0.0 | 0.0 |
| **7** | 44.0 | 12.5 | 0.0 | 1.0 | 2.0 | 3.0 | 0.0 |
| **8** | 36.0 | 62.5 | 0.0 | 0.0 | 3.0 | 1.0 | 0.0 |
| **9** | 16.0 | 87.5 | 1.0 | 1.0 | 15.0 | 0.0 | 0.0 |
| **10** | 12.0 | 25.0 | 0.0 | 0.0 | 19.0 | 1.0 | 0.0 |
| **11** | nd | nd | 0.0 | 0.0 | 18.0 | 2.0 | 0.0 |
| **12** | nd | nd | 0.0 | 0.0 | 24.0 | 0.0 | 0.0 |
|  |  |  |  |  |  |  |  |
| **∅ (Beach)** | 48.8 ± 29.9 | 43.8 ± 30.2 | 0.2 ± 0.4 | 0.3 ± 0.5 | 7.3 ± 8.9 | 0.8 ± 1.0 | 0.0 ± 0.0 |
| **∅ (Region)** | 46.3 ± 29.3 | | 0.3 ± 0.4 | | 4.1 ± 7.0 | | 0.0 ± 0.0 |
