# Supplementary material for: Daily accumulation rates of floating debris and attached biota on continental and oceanic island shores in the SE Pacific: testing predictions based on global models
Source: PeerJ. 2023 Jul 27;11:e15550. doi: 10.7717/peerj.15550 (PMC10387232; doi:10.7717/peerj.15550)
Supplement: Table S4 — All, all items; pel, items with pelagic epibionts only. N, number of items considered. Main categories (bold) and subcategories with a minimum frequency of 5% at any sampling site are shown. Subcategories with a frequency of <5% were pooled. * no items with pelagic epibionts were found during quantitative daily samplings on Choros beach. [file peerj-11-15550-s004.docx]

**Table S4**. **Detailed composition of AMD found during quantitative daily samplings within the defined sampling transect of each beach.** All = all items, pel = items with pelagic epibionts only. N = number of items considered. Main categories (bold) and subcategories with a minimum frequency of 5% at any sampling site are shown. Subcategories with a frequency of < 5% were pooled. * no items with pelagic epibionts were found during quantitative daily samplings on Choros beach.

|  | **Anakena** | | **Ovahe** | | **Mar Brava South** | | **Mar Brava North** | | **Ritoque** | | **Maitencillo** | | **Choros** | |  |
| --- | --- | --- | --- | --- | --- | --- | --- | --- | --- | --- | --- | --- | --- | --- | --- |
|  | **all** | **pel** | **all** | **pel** | **all** | **pel** | **all** | **pel** | **all** | **pel** | **all** | **pel** | **all** | **pel** |  |
| Number of items (N) | (499) | (122) | (240) | (35) | (887) | (2) | (449) | (4) | (5677) | (67) | (2758) | (9) | (949) | 0* |  |
|  | **[%]** | **[%]** | **[%]** | **[%]** | **[%]** | **[%]** | **[%]** | **[%]** | **[%]** | **[%]** | **[%]** | **[%]** | **[%]** | **[%]** |  |
| **Hard Plastics (∑)** | **69.9** | **95.4** | **70.0** | **100.0** | **9.4** | **50.0** | **14.9** | **25.0** | **20.0** | **32.9** | **20.9** | **33.3** | **5.5** | **x** |  |
| Fragments (except PET) | 66.0 | 95.4 | 68.4 | 100.0 | 5.9 | x | 7.7 | x | 9.1 | 20.9 | 8.5 | 11.1 | 3.8 | x |  |
| Caps and lids | 3.5 | x | 1.6 | x | 2.3 | x | 4.9 | x | 7.3 | 9.0 | 8.3 | 11.1 | 0.8 | x |  |
| Items, miscellaneous | 0.4 | x | x | x | 1.2 | 50.0 | 2.3 | 25.0 | 3.6 | 3.0 | 4.1 | 11.1 | 0.9 | x |  |
| **Ropes (∑)** | **27.0** | **4.6** | **28.4** | **x** | **54.4** | **x** | **44.3** | **50.0** | **3.5** | **6.0** | **3.6** | **22.2** | **40.7** | **x** |  |
| **Thin Plastics (∑)** | **3.0** | **x** | **0.8** | **x** | **30.1** | **x** | **14.7** | **x** | **59.9** | **49.3** | **53.1** | **44.4** | **28.6** | **x** |  |
| Packaging | 1.8 | x | 0.4 | x | 27.0 | x | 11.0 | x | 49.4 | 41.8 | 40.6 | 33.3 | 15.3 | x |  |
| Food containers, cups & cutlery, single use | 1.0 | x | 0.4 | x | 0.9 | x | 0.9 | x | 7.0 | 4.5 | 9.5 | x | 0.8 | x |  |
| Bags | 0.2 | x | x | x | 2.2 | x | 2.8 | x | 3.5 | 3.0 | 3.0 | 11.1 | 12.5 | x |  |
| **Other Plastics (∑)** | **x** | **x** | **x** | **x** | **4.6** | **x** | **8.5** | **x** | **10.2** | **4.5** | **8.5** | **x** | **17.7** | **x** |  |
| Foams | x | x | x | x | 0.9 | x | 5.2 | x | 5.4 | 1.5 | 4.0 | x | 0.7 | x |  |
| Fragments, PET | x | x | x | x | 0.2 | x | x | x | 0.1 | x | x | x | 11.4 | x |  |
| Other | x | x | x | x | 3.5 | x | 3.3 | x | 4.7 | 3.0 | 4.5 | x | 5.6 | x |  |
| **Other, Mix (∑)** | **0.2** | **x** | **0.8** | **x** | **1.4** | **50.0** | **17.5** | **25.0** | **6.3** | **7.5** | **13.9** | **x** | **8.2** | **x** |  |
| Processed wood | x | x | x | x | 0.5 | 50.0 | 0.7 | 25.0 | 2.7 | 1.5 | 8.7 | x | 0.4 | x |  |
| Organic/ Food rests | x | x | x | x | x | x | 12.6 | x | 0.6 | x | 0.2 | x | 0.1 | x |  |
| Clothing & shoes | x | x | x | x | 0.2 | x | 0.7 | x | 0.4 | 3.0 | 0.9 | x | 6.3 | x |  |
| Other | 0.2 | x | 0.8 | x | 0.7 | x | 3.5 | x | 2.6 | 3.0 | 4.1 | x | 1.4 | x |  |
| **∑** | **100** | **100** | **100** | **100** | **100** | **100** | **100** | **100** | **100** | **100** | **100** | **100** | **100** | **x** |  |
