# Supplementary material for: Daily accumulation rates of floating debris and attached biota on continental and oceanic island shores in the SE Pacific: testing predictions based on global models
Source: PeerJ. 2023 Jul 27;11:e15550. doi: 10.7717/peerj.15550 (PMC10387232; doi:10.7717/peerj.15550)
Supplement: Table S5 — (A) All AMD items, (B) only items with pelagic epibionts. Only items found during quantitative daily samplings within the defined sampling sections were included (no items from opportunistic samplings). [file peerj-11-15550-s005.docx]

**Table S5. Results of similarity percentage analysis (SIMPER**), based on Bray-Curtis similarities, showing within-group similarities in AMD composition for the four sampled regions. (A) All AMD items, (B) only items with pelagic epibionts. Only items found during quantitative daily samplings within the defined sampling sections were included (no items from opportunistic samplings).

**A) All AMD items**

| **Litter**  **category** | **Abundance (mean)** | **Similarity (mean)** | **Similarity * SD^-1^** | **Contribution [%]** | **Cumulative Contribution [%]** |
| --- | --- | --- | --- | --- | --- |
| **Group Oceanic_Rapa Nui: Average similarity: 80.96** | | | | | |
| **Hard plastics** | 66.95 | 58.03 | 3.96 | 71.67 | 71.67 |
| **Ropes** | 31.05 | 22.41 | 2.30 | 27.68 | 99.36 |
| **Group Continental_South: Average similarity: 72.74** | | | | | |
| **Ropes** | 50.96 | 43.00 | 3.49 | 59.12 | 59.12 |
| **Thin plastics** | 22.83 | 15.36 | 1.98 | 21.12 | 80.24 |
| **Hard plastics** | 12.46 | 8.05 | 1.81 | 11.06 | 91.30 |
| **Group Continental_Center: Average similarity: 72.98** | | | | | |
| **Thin plastics** | 58.38 | 48.91 | 3.14 | 67.01 | 67.01 |
| **Hard plastics** | 20.50 | 12.36 | 1.41 | 16.94 | 83.95 |
| **Other plastics** | 9.88 | 5.92 | 1.74 | 8.10 | 92.06 |
| **Group Continental_North: Average similarity: 74.83** | | | | | |
| **Ropes** | 32.50 | 26.74 | 4.32 | 35.73 | 35.73 |
| **Thin plastics** | 28.08 | 23.75 | 3.51 | 31.74 | 67.47 |
| **Other plastics** | 20.33 | 16.85 | 4.65 | 22.52 | 89.98 |
| **Other, mix** | 11.17 | 5.15 | 1.16 | 6.88 | 96.87 |

**B) Items with pelagic epibionts only**

| **Litter**  **category** | **Abundance (mean)** | **Similarity (mean)** | **Similarity * SD^-1^** | **Contribution [%]** | **Cumulative Contribution [%]** |
| --- | --- | --- | --- | --- | --- |
| **Group Oceanic_Rapa Nui: Average similarity: 93.79** | | | | | |
| **Hard plastics** | 96.37 | 93.26 | 10.80 | 99.44 | 99.44 |
| **Group Continental_South: Average similarity: 20.00** | | | | | |
| **Ropes** | 33.33 | 6.67 | 0.26 | 33.33 | 33.33 |
| **Other, Mix** | 33.33 | 6.67 | 0.26 | 33.33 | 66.67 |
| **Hard plastics** | 33.33 | 6.67 | 0.26 | 33.33 | 100.00 |
| **Group Continental_Center: Average similarity: 34.98** | | | | | |
| **Thin plastics** | 44.22 | 20.89 | 0.72 | 59.73 | 59.73 |
| **Hard plastics** | 27.73 | 11.72 | 0.64 | 33.51 | 93.24 |
