# Supplementary material for: Daily accumulation rates of floating debris and attached biota on continental and oceanic island shores in the SE Pacific: testing predictions based on global models
Source: PeerJ. 2023 Jul 27;11:e15550. doi: 10.7717/peerj.15550 (PMC10387232; doi:10.7717/peerj.15550)
Supplement: Table S6 — Frequency of occurrence of each taxon amongst all fouled items. yes, found in opportunistic sampling only (=not found during quantitative samplings). (yes) = entangled, but not attached. n.i. = not (further) identified. Habitat: ben = benthic, pel = pelagic. # = found on negatively buoyant items only. ∑taxa per beach = minimum number of distinguishable taxa per sampled beach. [file peerj-11-15550-s006.docx]

**Table S6.** Species list. Frequency of occurrence of each taxon amongst all fouled items. yes = found in opportunistic sampling only (= not found during quantitative samplings). (yes) = entangled, but not attached. n.i. = not (further) identified. Habitat: ben = benthic, pel = pelagic. ^#^ = found on negatively buoyant items only. ∑ taxa per beach = minimum number of distinguishable taxa per sampled beach. *almost certainly *Escharina brongniatiana* d’Orbigny, 1842, but, in the absence of more characters to confirm its separate identity, we defer using the combination *Celleporella brongniartiana*.

|  | **Ana**  (N=129) | **Ova**  (N=39) | **MBS**  (N=17) | **MBN**  (N=15) | **Rit**  (N=346) | **Mait**  (N=69) | **Cho**  (N=540) | **habitat** |
| --- | --- | --- | --- | --- | --- | --- | --- | --- |
| **Phylum Foraminifera - Class Globothalamea – Order Rotallida (∑)** | x | x | x | x | **1.8** | **1.4** | x |  |
| Rosalinidae, *Rosalina globularis* d’Orbigny, 1826 | x | x | x | x | 1.2 | 1.4 | x | ben |
| Planulinidae, *Planulina ariminensis* d’Orbigny, 1826 | x | x | x | x | yes | x | x | ben |
| **Phylum Bryozoa (∑)** | **93.0** | **89.7** | **17.6** | **20.0** | **56.4** | **26.1** | **1.3** |  |
| Encrusting bryozoa, n.i. (not Membraniporidae) | x | x | x | x | 4.0 | 5.8 | 0.9 | x |
| **Class Gymnolaemata** **– Order Cheilostomatida (∑)**  Cheilostomatida n.i. | **93.0**  x | **89.7**  x | **17.6**  x | **20.0**  6.7 | **52.9**  3.5 | **20.3**  x | **0.4**  x | x  x |
| **Suborder Flustrina (∑)**  Flustrina n.i. | **x**  x | **x**  x | **5.9**  5.9 | **13.3**  yes | **16.2**  4.0 | **10.1**  4.3 | **0.4**  0.2 | x  x |
| **Superfamily Buguloidea (∑)**  Bugoloidea, n.i.  Bugulidae, *Bugula neritina*  *Bugulina cf. flabellata* | **x**  x  x  x | **x**  x  x  x | **x**  x  x  x | **x**  x  x  x | **0.3**  0.3  x  x | **yes**  x  yes  x | **yes**  x  yes  yes | x  x  ben  ben |
| **Superfamily Calloporoidea (∑)**  Calloporidae, *Cauloramphus* sp.  Chaperiidae, *Chaperia* sp.  Ellisinidae, *Ellisina* sp.  Foveolariidae, *Odontionella* sp. | **X**  x  x  x  x | **x**  x  x  x  x | **5.9**  yes  5.9  x  x | **x**  x  x  x  x | **4.6**  0.3  4.0  0.3  0.3 | **2.9**  x  2.9  x  x | **yes?**  Yes?  x  x  x | x  ben  ben  ben  ben |
| **Superfamiliy Hippothooidea**, Hippothoidae **(∑)**  Hippothoidae, n.i.  *Celleporella hyalina* auctt./ *C*. *brongniartiana** | **x**  x  x | **x**  x  x | **yes**  yes  x | **yes**  yes  x | **7.5**  7.2  0.3 | **1.4**  1.4  x | **0.2**  0.2  x | x  ben  ben |
| **Superfamily Lepralielloidea**, Exochellidae, *Exochella* sp. | **x** | **x** | **yes** | **x** | **0.9** | **x** | **x** | ben |
| **Superfamily Microporoidea**, Microporidae, *Andreella megapora* | **x** | **x** | **x** | **x** | **yes** | **x** | **x** | ben |
| **Superfamily Schizoporelloidea (∑)**  Schizoporelloidea, n.i.  Fenestrulinidae, *Fenestrulina* sp  Hippaliosinidae, *Hippaliosina dorbignyana*  Teuchoporidae, *Lagenicella variabilis* | **x**  x  x  x  x | **x**  x  x  x  x | **5.9**  5.9  yes  x  x | **13.3**  13.3  x  x  x | **2.3**  1.7  0.3  0.3  x | **5.8**  1.4  1.4  1.4  1.4 | **x**  x  x  x  x | x  x  ben  ben  ben |
| **Suborder Membraniporina, Superfamily Membraniporoidea (∑)**  Electridae, n.i.  *Conopeum* sp.  Membraniporidae, *Jellyella eburnea*  *Membranipora* sp. | **93.0**  x  x  93.0  x | **89.7**  x  x  89.7  x | **yes**  yes  x  x  x | **yes**  yes  x  x  x | **37.9**1.4  0.3  x  36.1 | **10.1**  x  x  x  10.1 | **x**  x  x  x  x | x  x  ben  pel  ben |
| **Class Stenolaemata** **– Order Cyclostomatida (∑)** | **x** | **x** | **x** | **x** | **0.8** | **x** | **x** |  |
| **Suborder Tubuliporina (∑)**  Plagioeciidae, *Plagioecia* sp.  Tubuliporidae, *Tubulipora* sp. | **x**  x  x | **x**  x  x | **x**  x  x | **x**  x  x | **0.8**  0.3  0.6 | **x**  x  x | **x**  x  x | x  ben  ben |
| **Suborder Rectangulata**, Lichenoporidae, *Disporella* sp. | x | x | x | x | **yes** | x | x | ben |
| **Phylum Cnidaria - Class Anthozoa** | **3.1** | **x** | **x** | **x** | **1.4** | **x** | **yes** |  |
| Order Actiniaria (∑)  Actiniidae, *Anthopleura cf. hermaphroditica* | x  x | x  x | x  x | x  x | 1.4  0.3 | x  x | yes  x | x  ben |
| *Phymactis papillosa* | x | x | x | x | 1.2 | x | x | ben |
| Sagartiidae, *Paranthus niveus* | x | x | x | x | yes | x | x | ben |
| Order Scleractinia, Pocilloporidae, *Pocillopora* sp. | 3.1 | x | x | x | x | x | x | ben |
| **Phylum Cnidaria - Class Hydrozoa** | **9.3** | **5.1** | **35.3** | **20.0** | **18.7** | **14.5** | **0.4** |  |
| Hydrozoa, n.i. | 9.3 | 5.1 | 35.3 | 20.0 | 18.7 | 14.5 | 0.4 | x |
| Order Leptothecata, n.i. | x | x | x | x | 1.4 | x | x | x |
| Plumulariidae, *Plumularia setacea* | x | x | (yes?) | (yes?) | x | x | (yes?) | ben |
| Sertulariidae n.i. | x | x | (yes) | (yes) | x | (yes) | (yes) | x |
| *Amphisbetia operculata* | x | x | (yes) | (yes) | x | x | x | ben |
| *Sertularella* sp. | x | x | x | x | (yes) | x | x | ben |
| **Phylum Nematoda** | x | x | x | yes | x | x | x |  |
| **Phylum Mollusca - Class Bivalvia** | **0.8** | x | **17.6** | **6.7** | **6.1** | **40.6** | **99.1** |  |
| Order Mytilida, Mytilidae, n.i.  Order Ostreida, Ostreidae, n.i. | x  0.8 | x  x | 17.6  x | 6.7  x | 6.1  x | 40.6  x | 99.1  x | ben  ben |
| **Phylum Mollusca - Class Gastropoda** | x | x | **5.9** | **6.7** | **2.0** | **2.9** | **x** |  |
| Nudibranchia (∑)  egg masses, n.i. | x  x | x  x | 5.9  5.9 | x  x | 0.9  0.9 | x  x | x  x | x  x |
| Fionidae, *Fiona pinnata* | x | x | 5.9 | x | x | x | x | pel |
| Littorinimorpha, Calyptraeidae, *Trochita trochiformis* | x | x | x | x | 0.3**^#^** | **2.9** | x | ben |
| Siphonariida, Siphonariidae, S*iphonaria lessonii* | x | x | x | x | 0.3 | x | x | ben |
| Lottiidae, *Scurria* sp. / *Scurria variabilis* | x | x | x | x | 0.3 | x | x | ben |
| *Lottia orbignyi* | x | x | x | x | 0.3 | x | x | ben |
| Gastropoda, n.i. | x | x | x | 6.7 | x | x | x | x |
| **Phylum Annelida - Class Polychaeta** | **0.8** | **10.3** | **11.8** | **12.5** | **10.4** | **8.7** | **0.6** |  |
| Polychaeta, n.i. | x | x | yes | yes | 1.4 | x | x | x |
| Subclass Sedentaria, Order Sabellida (∑)  Serpulidae, Spirorbinae | 0.8  0.8 | 10.3  10.3 | 5.9  5.9 | 20.0  20.0 | 9.2  8.1 | 8.7  8.7 | yes  yes | x  ben |
| Serpulidae, not Spirorbinae | x | 5.1 | x | x | 0.3 | x | yes | ben |
| Sabellidae | x | x | x | x | x | x | yes | ben |
| Sabellariidae | x | x | yes | x | 0.9 | yes | 0.2^#^ | ben |
| Subclass Errantia (∑)  Order Phyllodocida, Nereididae, n.i., possibly *Platynereis australis*  Phyllodocidae, n.i.  Polinoidae, *Harmothoe* sp? | x  x  x  x | x  x  x  x | 5.9^#^  5.9^#^  x  x | x  x  x  x | 0.3  x  x  x | x  x  x  x | 0.4  yes  yes  yes | x  ben  ben  ben |
| Order Spionida, Spionidae, n.i. | x | x | x | x | 0.3 | x | x | ben |
| **Phylum Arthropoda - Class Malacostraca** | x | x | 5.9 | x | **1.4** | **4.3** | yes |  |
| Order Isopoda, Sphaeromatidae | x | x | 5.9 | x | x | x | x | x |
| Order Amphipoda, Caprellidae, *Deutella venenosa* | x | x | x | x | yes | x | yes | ben |
| *Caprella penantis f. gibbosa* sensu Mayer | x | x | x | x | yes | x | yes | ben |
| Ischyroceridae, *Jassa cf. marmorata* | x | x | x | x | yes | x | x | ben |
| Stenothoidae, *Stenothoe* sp. | x | x | x | x | yes | x | x | ben |
| Order Decapoda, Infraorder Brachyura, n.i. | x | x | x | x | 1.2 | 2.9 | yes | x |
| Porcellanidae, n.i. | x | x | x | x | 0.3 | 1.4 | x | x |
| **Phylum Arthropoda – Class Pycnogonida** | **x** | **x** | **x** | **x** | **x** | **x** | **yes** |  |
| **Phylum Arthropoda - Class Thecostraca** | **7.0** | **2.6** | **10.0** | **31.3** | **48.1** | **40.6** | **2.2** |  |
| **Order Balanomorpha (∑)**  Balanomorpha, n.i. | **x**  x | **x**  x | **3.3**  3.3 | **6.3**  x | **15.9**  5.2 | **15.9**  x | **2.2**  yes | x  x |
| Balanidae, n.i. | x | x | x | 6.3 | 5.2 | 2.9 | 0.2 | x |
| *Balanus laevis* | x | x | x | x | 4.6 | 7.2 | 2.2 | ben |
| *Austromegabalanus psittacus* | x | x | x | x | x | 1.4 | yes | ben |
| *Notobalanus flosculus* | x | x | x | x | 0.6 | x | yes | ben |
| Chthamalidae, n.i. | x | x | x | x | 1.2 | 1.4 | x | x |
| *Notochthamalus scabrosus* | x | x | yes | x | 2.9 | 1.4 | x | ben |
| **Order Verrucomorpha**, Verrucidae, *Verruca laevigata* | **x** | **x** | **x** | **yes** | **2.6** | **2.9** | **yes** | ben |
| **Order Scalpellomorpha**, Lepadidae **(∑)**  *Lepas*, n.i. | **7.0**  5.4 | **2.6**  2.6 | **11.8**  5.9 | **26.7**  6.7 | **19.3**  6.6 | **13.0**  8.7 | **yes**  yes | x  pel |
| *Lepas anatifera* | 1.6 | x | x | x | x | x | x | pel |
| *Lepas pectinata* | x | x | 5.9 | 20.0 | 12.1 | 4.3 | yes | pel |
| *Lepas australis* | x | x | yes | 6.7 | 1.2 | x | x | pel |
| **Phylum Chordata - Class Ascidiacea** | **x** | **x** | **x** | **x** | **x** | **x** | **yes** |  |
| Order Stolidobranchia, Pyuridae, *Pyura chilensis* | x | x | x | x | x | x | yes | ben |
| Order Phlebobranchia, Cionidae, *Ciona robusta* | x | x | x | x | x | x | yes | ben |
| Colonial ascidian, ni | x | x | x | x | x | x | yes | x |
| **Encrusting, calcareous structures (bacteria?)** | 1.6 | 10.3 | x | yes? | yes | x | x |  |
| **∑ taxa per beach** | **7** | **6** | **16** | **14** | **43** | **19** | **27** |  |
| **Species richness (∑ taxa * item^-1^)** | **1.2**  **±0.4** | **1.2**  **±0.6** | **1.4**  **±0.9** | **1.4**  **±0.8** | **1.7**  **±1.3** | **1.5**  **±0.9** | **1.1**  **±0.9** |  |
