# Supplementary figures and images for: Daily accumulation rates of floating debris and attached biota on continental and oceanic island shores in the SE Pacific: testing predictions based on global models
Source: PeerJ. 2023 Jul 27;11:e15550. doi: 10.7717/peerj.15550 (PMC10387232; doi:10.7717/peerj.15550)

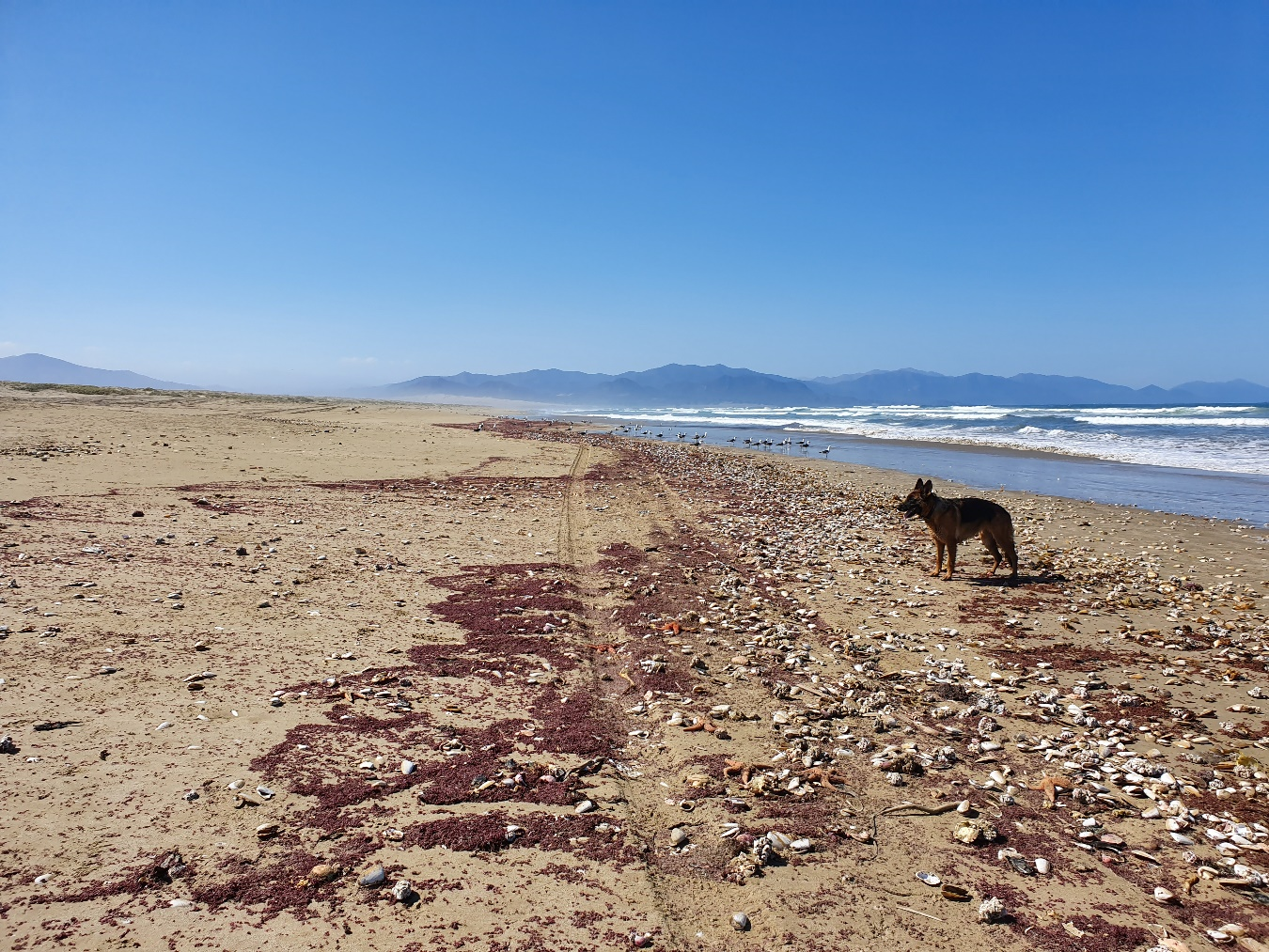

Supplement: Figure S1 [file peerj-11-15550-s007.png]

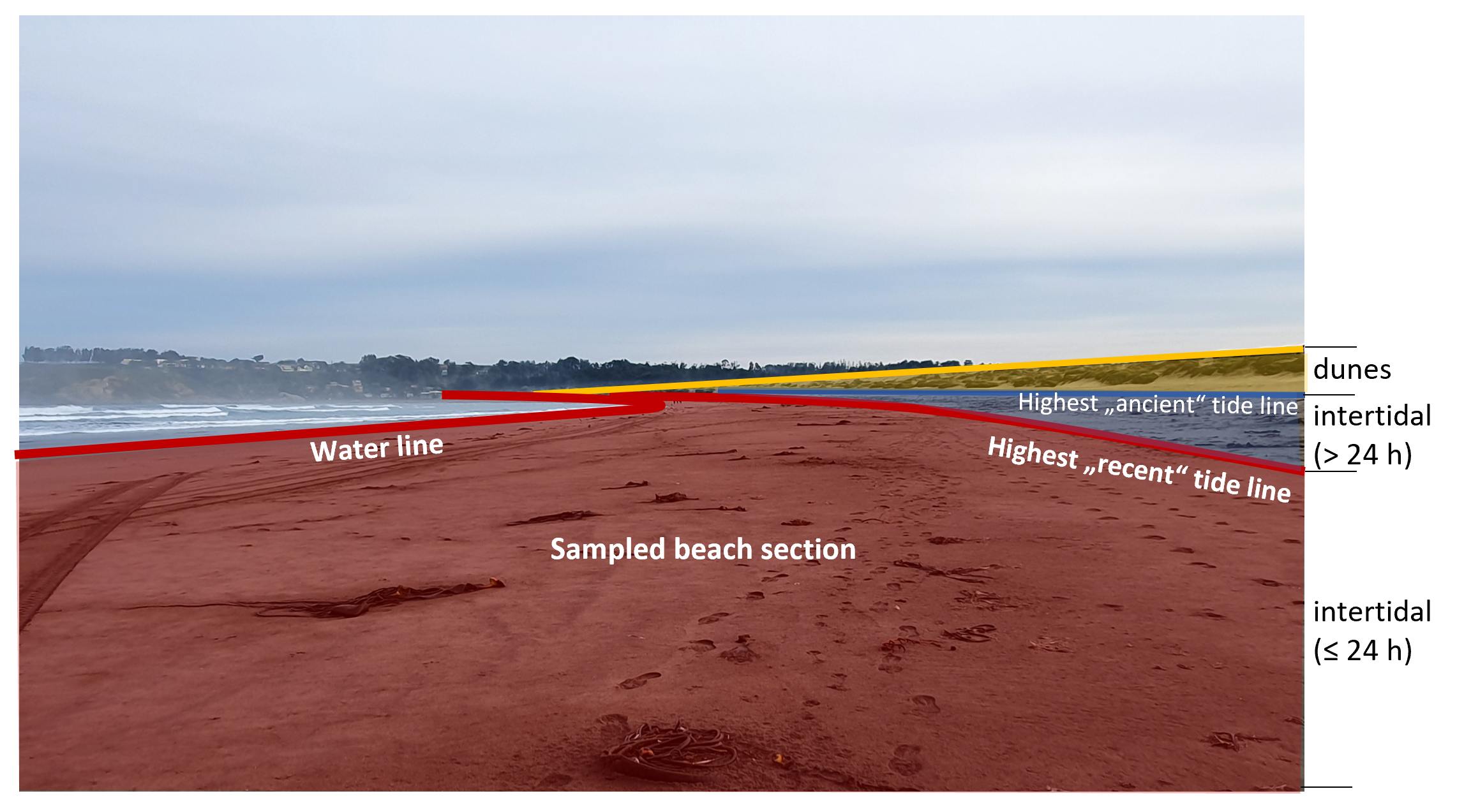

Supplement: Figure S2 — Red: “Recent” intertidal with tidelines from the last 24 hours (=sampled beach section). Blue: “Ancient” intertidal, with tidelines older than 24 hours (not sampled). Yellow: Dunes (not sampled). [file peerj-11-15550-s008.png]

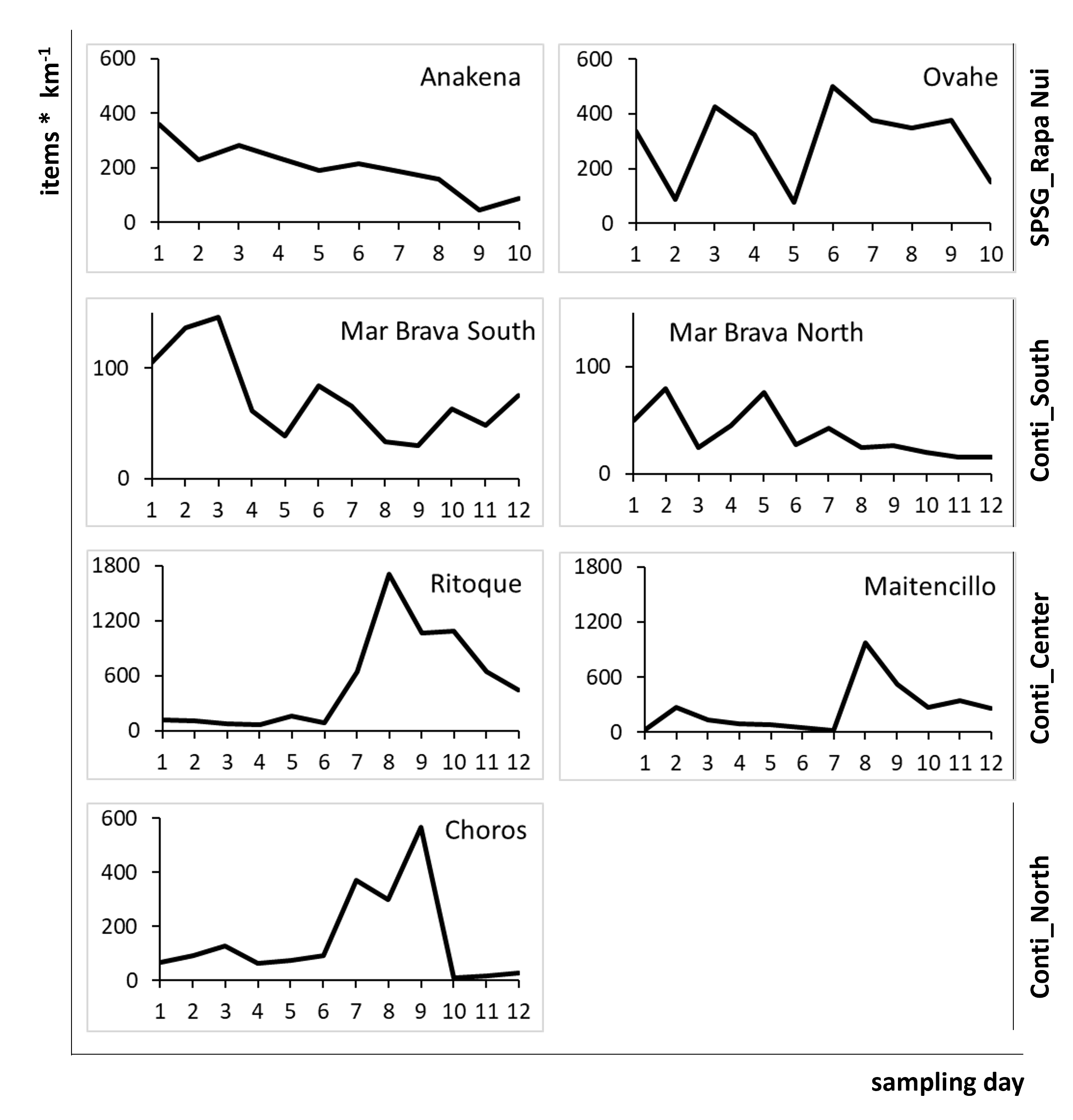

Supplement: Figure S3 — SPSG, South Pacific Subtropical Gyre; Conti, Continental. [file peerj-11-15550-s009.png]

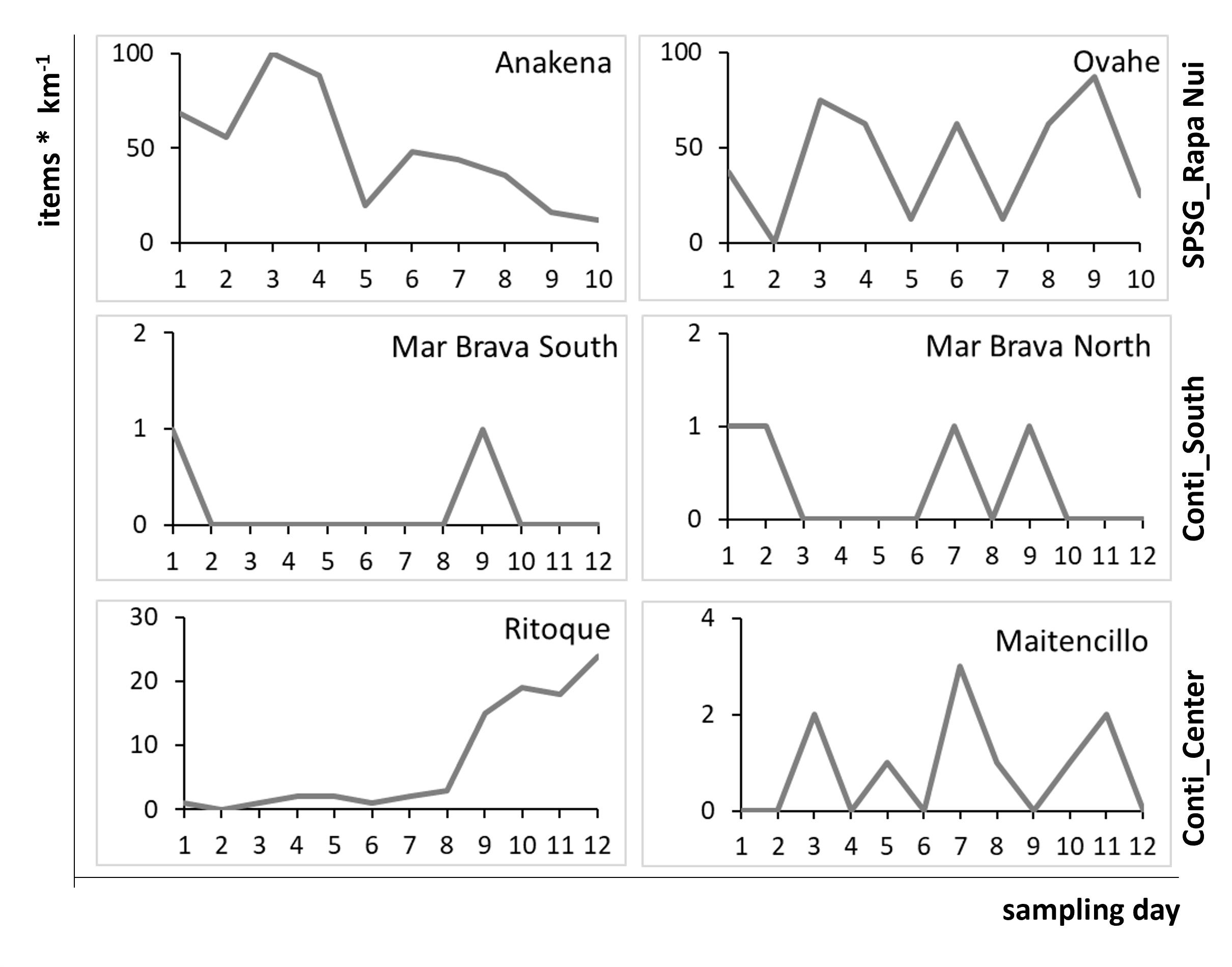

Supplement: Figure S4 — SPSG, South Pacific Subtropical Gyre; Conti, Continental. No litter items carrying pelagic epibionts were found during quantitative daily samplings on Choros beach in the northern continental region. [file peerj-11-15550-s010.png]

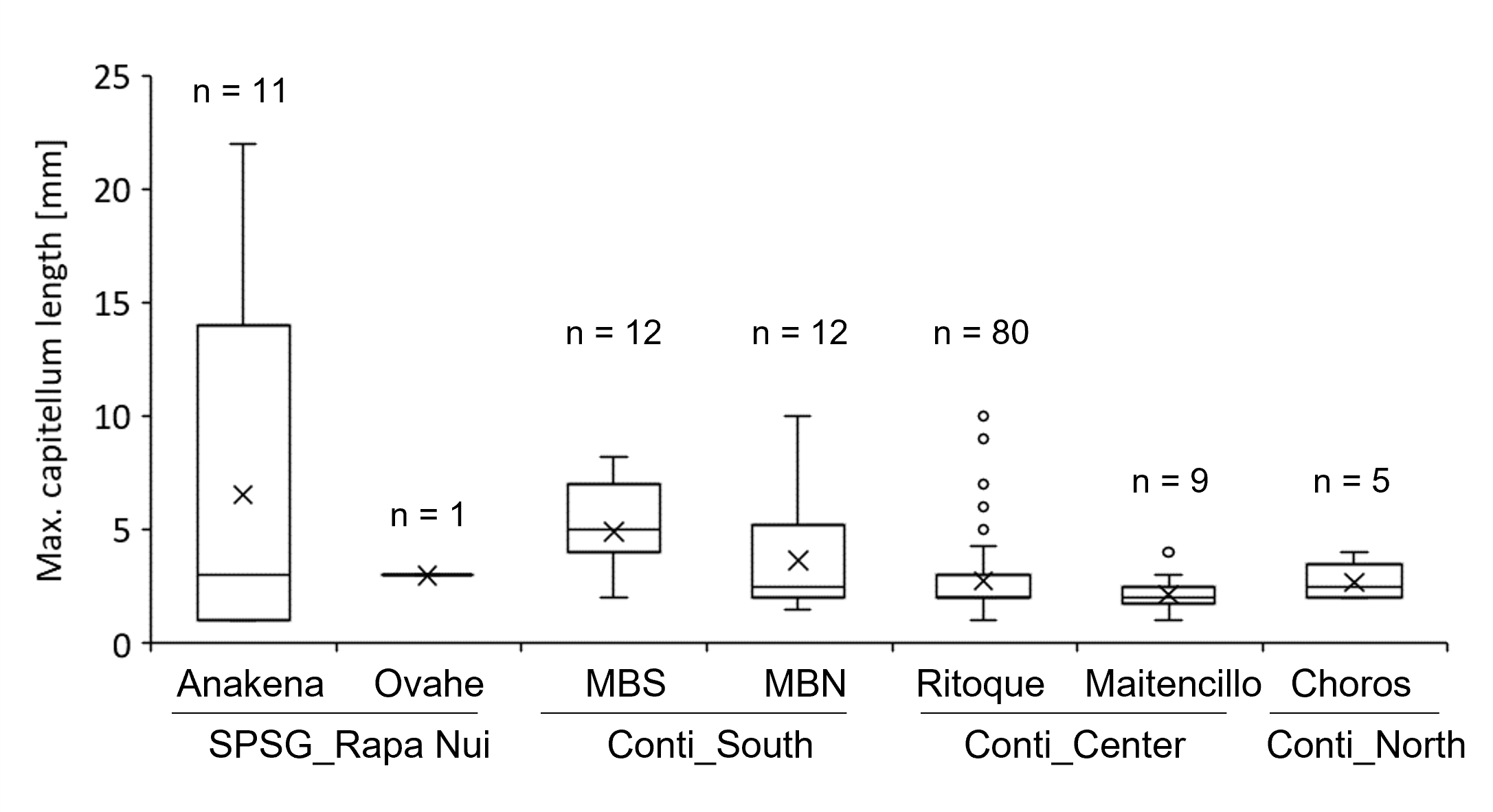

Supplement: Figure S5 — Samples from quantitative and opportunistic samplings are pooled. n, number of individuals considered at each beach. Boxplot shows average, median, quartiles and outliers for each site, based on the largest individual of each identifiable species of each raft. SPSG, South Pacific Subtropical Gyre. Conti, Continental; MBS, Mar Brava South; MBN, Mar Brava North. [file peerj-11-15550-s011.png]

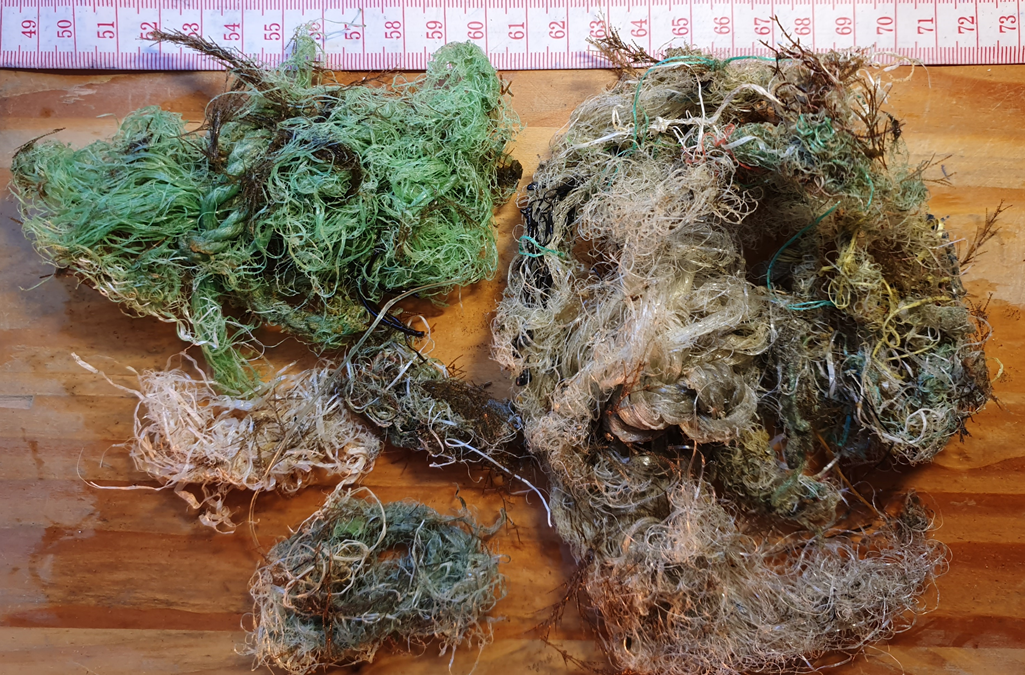

Supplement: Figure S6 — White bundle on the left is a fragment of a woven plastic bag, rather than a rope bundle. [file peerj-11-15550-s012.png]
