# Supplementary material for: Daily accumulation rates of floating debris and attached biota on continental and oceanic island shores in the SE Pacific: testing predictions based on global models
Source: PeerJ. 2023 Jul 27;11:e15550. doi: 10.7717/peerj.15550 (PMC10387232; doi:10.7717/peerj.15550)
Supplement: Text S1 — Additional information about sampled sites and regions, as well as the sampled beach sections. [file peerj-11-15550-s013.docx]

**SUPPLEMENT**

Daily accumulation rates of floating debris and attached biota on continental and oceanic island shores in the SE Pacific: Testing predictions based on global models.

Sabine Rech^1,2^, René Matías Arias^1^, Simón A Heru Vadell Teave^1^, Dennis Gordon ^3^, Martin Thiel^1,2,4^

^1^ Facultad de Ciencias del Mar, Universidad Católica del Norte, Coquimbo, Chile

^2^ Center for Ecology and Sustainable Management of Oceanic Islands (ESMOI), Departamento de Biología Marina, Facultad de Ciencias del Mar, Universidad Católica del Norte, Coquimbo, Chile

^3^ National Institute of Water and Atmospheric Research (NIWA), Kilbirnie, Wellington, New Zealand

^4^ Centro de Estudios Avanzados en Zonas Áridas (CEAZA), Avenida Ossandón 877, Coquimbo, Chile

Corresponding author: Sabine Rech^1,2^

Email address: sabine.rech@ucn.cl, sabinerech01@gmail.com

**SUPPLEMENTAL TEXT**

**Material & methods**

**Sampling sites**

Rapa Nui is a very remote, small island (area = 163 km^2^; population density = 23.1 inhabitants km^-2^ as of 2017; INE, 2017), located in the centre of the South Pacific Subtropical Gyre (SPSG). Usually overcrowded with tourists from all over the world, it was completely closed off during the COVID19 pandemic when samplings took place, limiting beach use to local residents. Anakena is the main beach used for leisure activities as it is bigger (about 250 m long with a wide dune area) and easily accessible by car. It is situated in a relatively sheltered bay with (usually) calm waters. Ovahe, on the other hand, is a very short (80m) and narrow sandy beach, limited by rocks and cliffs, which is frequently flooded during high tides. Rapa Nui is known to receive large amounts of floating AMD from the SPSG, which are mainly attributed to sources from continental Chile and industrial fisheries (Van Gennip et al., 2019). There are no rivers or bigger settlements on the island.

Chiloé island in the south of Chile, is separated from the mainland by the Sea of Chiloé to the East and the Chacao Channel in the North. The island is scarcely populated (18.3 inhabitants km^-2^; INE, 2017), with main settlements and economic activities (aquaculture; SUBPESCA 2023) concentrated on the sheltered “inner” coast. Mar Brava beach is located on the exposed “outer” coast, facing the South Pacific Ocean, with no major settlements or economic activities. During our samplings, in austral autumn of 2021, tourism and general movement were strongly restricted on the island due to the COVID19 pandemic and the main beach-going season, even for local residents, had already passed. Mar Brava has a total length of about 8 km, however, due to their different characteristics, the northern (Mar Brava North, MBS) and southern part (Mar Brava South, MBS) were sampled separately and treated as two different sites in our study. While both are exposed to the open ocean to the west, the southern part is protected towards the main wind and current direction by a headland and is under the influence of the Pudeyi River mouth, located about 1 km south of our sampling transect. There, the beach was strongly erosional, with a narrow intertidal area and a very steep slope (up to 90°) towards the elevated dunes. The northern part of Mar Brava beach, on the contrary, has no protective structures towards the main current direction, is situated further away from the river mouth (5 km distance) and is characterized by a wide intertidal area and a gentle slope towards the low-lying dunes.

Ritoque and Maitencillo are located in the central Chilean Valparaiso province (population density 262.3 inhabitants km^-2^; INE, 2017) in vicinity of two bigger cities, Valparaiso and Viña del Mar, as well as Valparaiso international port. The area is a prominent destination for tourists from the close-by metropolitan region around the capital Santiago, which concentrates about 40% of the Chilean population and constitutes the country’s economic center. Despite the restrictions related to the COVID19-pandemic, the region´s beaches were frequently visited during the sampling period, as many residents of Santiago`s high-income neighbourhoods moved to their summer houses on the coast. In the popular seaside resort Maitencillo, our sampling transect was located on a beach strip locally known as “Playa Aguas Blancas”, with a total length of 4 km. The westward-facing beach is rather wide and flat, being limited by parking lots, buildings and the main avenue, constructed in the former dune area. We have not observed any official beach cleaning activities, however, occasionally, beach-goers would pick up some litter. Located 20 km (linear distance) further south, Ritoque beach is a long (14 km) and comparatively natural beach, with a moderate slope and an extensive area of elevated dunes. Our sampling section was located in its northern part, about 1 km from the access point and car park. During our samplings, the beach was much less frequently visited than Maitencillo and was mainly used by surfers and anglers. The south-westward-facing sampled section is situated 8 km downstream from the Aconcagua River mouth. Further south (100km from Ritoque), the bigger and heavily polluted Maipo River discharges into the sea, next to another international port (San Antonio) after passing the capital Santiago (see for example Rech et al., 2014).

Choros beach is a 15 km long beach in the northern-central Elqui Province (Coquimbo region), with a population density of 29.4 inhabitants km^-2^ (INE, 2017) situated 70-80 km (linear distance) north of the region´s main towns Coquimbo and La Serena, as well as the Elqui River mouth. There are no other major settlements along the region´s coast and, during our samplings during the Covid19 pandemic, the neighbouring village “Punta de Choros”, which is usually a popular spot for beach tourism and whale-watching, was virtually devoid of tourists. Consequently, Choros beach was visited only by artisanal fishers and collectors of stranded kelp. Similar to Ritoque beach in the central region, Choros has a moderate slope leading to a wide elevated area of dunes.

**Beach cleanings:**

Samplings took place during the COVID19 pandemic, when public life and tourism were strongly restricted in all sampled regions. While we have observed occasional litter-picking by beachgoers on Maitencillo beach, we are not aware of any larger or organized beach cleanings during the sampling period on the sampled continental beaches. On Rapa Nui, where the arrival of floating litter is a major concern and there is very high awareness of this problem among the local residents (see Kiessling et al., 2017), it is likely that clean-ups took place during the pandemic.

**Sampling section:**

The goal of the present study was the quantification of anthropogenic AMD arriving daily (i.e., within 24 hours) on the sampled beaches. Therefore, each morning, only the beach section that had been reached by the two last high tides was considered (= recent intertidal zone; Fig. S2). This zone reaches from the waterline to the highest recent tideline and varies in width and area, due to daily fluctuations in tidal and wave height. The highest “recent” tideline can be distinguished from more ancient high tide lines by containing relatively fresh organic and anorganic matter (e.g., macro- and microplastics, feathers, empty crustacean carapaces, macroalgae) deposited on the sand`s surface, while the latter are usually blurred through the impact of animals and beachgoers and often contain partially buried objects.

**Literature**

Instituto Nacional de Estadísticas (INE). Resultados Censo 2017. Available at http://resultados.censo2017.cl/ (accessed 20 March 2023).

Subsecretaría de Pesca y Acuicultura (SUBPESCA). Información de concesiones de acuicultura, listados de concesiones vigentes. Available at https://view.officeapps.live.com/op/view.aspx?src=https%3A%2F%2Fwww.subpesca.cl%2Fportal%2F619%2Farticles-92935_listado.xlsx&wdOrigin=BROWSELINK (accessed 23 March 2023).

Kiessling T, Salas S, Mutafoglu K, Thiel M. 2017. Who cares about dirty beaches? Evaluating environmental awareness and action on coastal litter in Chile. Ocean & Coastal Management, 137, 82-95. DOI: 10.1016/j.ocecoaman.2016.11.029

Rech S, Macaya-Caquilpán V, Pantoja J, Rivadeneira M, Jofre Madariaga D, Thiel M. 2014. Rivers as a source of marine litter - A study from the SE Pacific. Marine Pollution Bulletin 82: 1–2. DOI: 10.1016/j.marpolbul.2014.03.019

van Gennip SJV, Dewitte B, Garçon V, Thiel M, Popova E, Drillet Y, Ramos M, Yannicelli B, Bravo L, Ory N, Luna-Jorquera G, Gaymer C. 2019. In search for the sources of plastic marine litter that contaminates the Easter Island Ecoregion. Scientific Reports 9: 1–13. DOI: 10.1038/s41598-019-56012-x
